# Nonlinear associations of depression and sleep duration with cognitive impairment in older adults with hypertension: findings from a national survey

**DOI:** 10.3389/fnagi.2025.1579560

**Published:** 2025-07-15

**Authors:** Hui-Ying Fan, He-Li Sun, Yuan Feng, Qinge Zhang, Hua-Qing Xing, Qian-Hua Huang, Zhaohui Su, Teris Cheung, Chee H. Ng, Yu-Tao Xiang, Gang Wang

**Affiliations:** ^1^International Nursing School, Hainan Medical University, Haikou, Hainan, China; ^2^Unit of Psychiatry, Department of Public Health and Medicinal Administration, Faculty of Health Sciences, University of Macau, Macao SAR, China; ^3^Centre for Cognitive and Brain Sciences, University of Macau, Macao SAR, China; ^4^Beijing Key Laboratory of Mental Disorders, National Clinical Research Center for Mental Disorders and National Center for Mental Disorders, Beijing Anding Hospital, Capital Medical University, Beijing, China; ^5^School of Public Health, Southeast University, Nanjing, China; ^6^School of Nursing, Hong Kong Polytechnic University, Hong Kong SAR, China; ^7^Department of Psychiatry, The Melbourne Clinic and St Vincent's Hospital, University of Melbourne, Richmond, VIC, Australia

**Keywords:** older adults, hypertension, cognitive impairment, depression, sleep duration

## Abstract

**Objectives:**

Cognitive impairment is a major health concern in older adults with hypertension, and both depression and abnormal sleep duration are recognized as potential contributing factors. This study aimed to explore the nonlinear association of depression and sleep duration with cognitive impairment among older adults with hypertension.

**Methods:**

This cross-sectional study was based on the 2017–2018 wave of Chinese Longitudinal Healthy Longevity Survey. Depression and cognitive function were measured using the 10-item Center for Epidemiological Studies Short Depression Scale and Mini Mental State Examination, respectively. Univariate, binary logistic regression, and restricted cubic spline regression analyses were used to examine the associations between depression, sleep duration and cognitive impairment.

**Results:**

A total of 3,989 older adults with hypertension were included. The prevalence of depression and cognitive impairment were 28.1% (95%CI = 26.7–29.5%) and 10.1% (95%CI = 9.2–11.1%), respectively. After adjusting for confounding factors, a significant linear association (nonlinear *p* = 0.814) between depression and cognitive impairment risk was found, while a U-shaped nonlinear association was identified between sleep duration and cognitive impairment risk (*p* = 0.040). Both shorter (<6.6 h) and longer (>7.7 h) sleep duration per day were associated with higher cognitive impairment risk, with an inflection point at 7.3 h. The effect of sleep duration on cognitive impairment risk was more significant for participants with a higher (≥ 6 years) education level.

**Conclusion:**

This study highlights the importance of managing depression and optimizing sleep duration in addressing the risk of cognitive decline in older adults with hypertension.

## 1 Introduction

Hypertension is a highly prevalent chronic condition in older adults worldwide. With the rapidly aging population in China, there has been a substantial increase in the hypertension cases over past decades, with nearly half of adults over 65 affected (Tan et al., 2023; Wang et al., 2024). Hypertension is associated with specific cognitive impairment, particularly with regards to the development of vascular dementia and Alzheimer’s disease (Ungvari et al., 2021), thus increasing the burden on families and society (Lv et al., 2019). These links may be related to pathological changes in cerebral blood vessels induced by hypertension, contributing to cognitive decline. Such changes include remodeling (Scuteri et al., 2011a; Martinez-Lemus et al., 2009), dysregulation of blood flow and impaired cerebral perfusion (Pires et al., 2015; Capone et al., 2012). In a meta-analysis of 11 international studies, the estimated prevalence of mild cognitive impairment in hypertensive adults over age 60 was 28% [95% confidence interval (CI) = 23–33%] (Qin et al., 2021), highlighting the need to identify the associated risk factors in order to inform early intervention strategies and prevent cognitive impairment.

Depressive symptoms (hereafter depression) are common among older adults with hypertension, affecting up to 34% of this population (Yena et al., 2024). Multiple studies have consistently found a strong link between depression and cognitive impairment, with one analysis showing that depression nearly doubles the risk of poor cognitive performance among older adults [adjusted odds ratio (OR) = 2.25; 95%CI = 1.31–3.81] (Yao et al., 2024; Yin et al., 2024). Several studies have shown bidirectional relationships between depression and cognitive impairment (Guo et al., 2023; Huang et al., 2022; Yin et al., 2024). While many studies have explored the linear depression-cognitive impairment relationship, few have investigated their potential nonlinear association, especially in hypertensive older adults. One study revealed a J-shaped association between depression and cognitive decline in older adults in the US (Yao et al., 2024), while others only found linear relationships (Wu et al., 2024). Given such inconsistent findings, examining the potential nonlinear relationships could provide a deeper understanding of how depressive symptoms impact cognitive decline, potentially guiding targeted interventions for older adults with hypertension.

On the other hand, sleep duration is crucial for maintaining cognitive health. Adequate sleep supports cognitive processes, memory consolidation and the clearance of brain metabolic waste (Diekelmann, 2014; Wigren and Stenberg, 2015). Both shorter and longer sleep duration are known to be risk factors of hypertension (Pepin et al., 2014; Grandner et al., 2018) and are common among older adults with hypertension, often adversely impacting the overall quality of life (Kiełbasa et al., 2016; Uchmanowicz et al., 2019). One study found that self-reported sleep disturbances were linked to reduced cognitive performance among older adults with hypertension (Kohn et al., 2020). While other studies have found associations between sleep duration problems and cognitive impairment across various domains (e.g., recall, verbal fluency, and visual memory) (Henry et al., 2019; Warsame et al., 2023; Giannouli, 2017), there are contentious findings on the direction of such associations (Li et al., 2022; Ma et al., 2020; Albert, 2019; Suh et al., 2018). Certain studies identified U-shaped relationships, where both short and long sleep duration increase the risk of cognitive impairment (Ma et al., 2020; Li et al., 2022), whereas others found that only long sleep duration was associated with cognitive impairment, particularly among older adults (Zhang et al., 2022; Ding et al., 2024) and those with hypertension (Zhou et al., 2022). Thus, to clarify the trends or underlying mechanisms of the impact of sleep, the nonlinear association between sleep duration and cognitive impairment, particularly in older adults with hypertension, requires further investigation.

To address this gap, we examined the prevalence of cognitive impairment and depression among older adults with hypertension, and explore potential nonlinear associations between depression, sleep duration and cognitive impairment based on a national survey.

## 2 Methods

### 2.1 Study design and samples

This was a cross-sectional study based on the data from the 2017–2018 wave of the Chinese Longitudinal Healthy Longevity Survey (CLHLS) (Center for Healthy Aging Development Studies, 2020). The CLHLS is an ongoing, national cohort survey based on multi-stage random sampling across 23 of the 32 provinces in China (Zheng, 2020). The survey has been conducted every 2 to 3 years since 1998, with methodology details available in previous publications (Yu et al., 2021; Zeng et al., 2017). The inclusion criteria for participation in this study included: (1) participants aged 65 or older (Bai et al., 2023; Rodda et al., 2011), (2) presence of hypertension according to records from the CLHLS database, and (3) available data for depression, cognitive function, sleep duration and other included variables in this study. Following previous research (Bai et al., 2023), individuals with severe cognitive impairment, such as dementia, were excluded from the study. Ethical approval for the CLHLS was granted by the Peking University Research Ethics Committee (No. IRB00001052-13074), and all participants provided written informed consent.

### 2.2 Measurements

Cognitive function was evaluated using the validated Chinese version of the Mini-Mental State Examination (MMSE) (Folstein et al., 1975; Katzman et al., 1988). The MMSE includes 24 items assessing six dimensions: Orientation (5 items), Registration (3 items), Naming (1 item), Attention and Calculation (5 items), Recall (3 items), and Language (7 items). Each item was scored as zero (wrong or unable to answer) or one (correct), except for the Naming item that was scored from zero to seven according to the number of correct responses. The total scores range from 0 to 30, with lower scores representing poorer cognitive function. Because MMSE scores can be affected by education, following previous studies (Wu et al., 2019; Liang et al., 2022), cognitive impairment was defined in this study using education-specific cutoffs: <18, ≤20 and ≤24 for participants with illiteracy, 1–6 years of primary education and more than 6 years of junior or higher education, respectively.

Depression was measured with the validated Chinese version of 10-item Center for Epidemiologic Studies Short Depression Scale (CESD-10) (Andresen et al., 1994; Chen and Mui, 2014). This scale assesses the frequency of occurrence of depressive symptoms in the previous week from 0 (‘rarely or none of the time; <1 day’) to 3 (‘most or all of the time; 5–7 days’). Items 5, 7 and 10 are reversely scored. The total score ranges from 0 to 30 with higher scores indicating more severe depressive symptoms. A cut-off score of 10 was applied to identify individuals having depression (Andresen et al., 1994).

Sleep duration was evaluated with a specific question “How many hours do you usually sleep?.” Socio-demographic covariates included variables such as gender, age, place of residence, education level, marital status, cohabitation with family, and perceived economic status. Physical function was measured using the 8-item Lawton Instrumental Activities of Daily Living (IADL) scale (Lawton and Brody, 1969; Tong and Man, 2002), with each item being rated from 1 (complete dependence) to 3 (complete independence). The total score ranges from 8 to 24, with higher scores indicating greater independence (Yang et al., 2023).

### 2.3 Statistical analysis

All statistical analyses were conducted using R (version 4.3.2) (R Development Core Team, 2020). Depression prevalence, sleep duration and other covariates were compared between groups with cognitive decline and no cognitive decline using chi-square and independent two-sample Wilcoxon rank sum test, as appropriate. Multivariable analysis was performed using binary logistic regression (enter method) to identify factors independently associated with cognitive impairment. Cognitive impairment served as the dependent variable, while variables showing significant group differences in univariable analyses were included as independent variables. Adjusted ORs with 95%CIs were computed to quantify the association strengths.

Restricted cubic spline (RCS) regression is widely used for characterizing dose–response relationships between continuous exposures and outcomes when nonlinearity is suspected (Croxford, 2016). The RCS curves, fitted with three knots, were used to investigate the potential nonlinear association between depression (as a continuous variable), sleep duration and cognitive impairment, after adjusting for covariates that showed significant differences in the multivariable analysis. Statistical significance was set at *p* < 0.05 (two-tailed) for all analyses.

## 3 Results

### 3.1 Participant characteristics

A total of 3,989 older adults with hypertension were included in this study. Their demographic and clinical characteristics are shown in Table 1. Among the participants, the majority were female (56.6%), had six or fewer years of education (74%), resided in urban areas (62.9%) and lived with their family (78.6%). The mean age was 81.80 [Standard Deviation (SD) = 10.42] years. The mean sleep duration was 7.23 (SD = 2.19) hours per day.

**Table 1:** Comparisons of socio-demographic data between cognitive impairment and non-cognitive impairment groups among older adults with hypertension.

Variable	Total (*N* = 3,989)		Non-cognitive impairment (*N* = 3,587)		Cognition impairment (*N* = 402)		Univariable analyses		
	*N*	%	*N*	%	*N*	%	*χ* ^2^	df	*p*
Female	2,259	56.6	2,009	56.0	250	62.2	5.624	1	**0.018**
Rural residence	1,481	37.1	1,335	37.2	146	36.3	0.125	1	0.723
Married	1,909	47.9	1,824	50.9	85	21.1	127.830	1	**<0.001**
Living with family	3,134	78.6	2,822	78.7	312	77.6	0.242	1	0.623
Junior education or above (>6 years)	1,037	26.0	953	26.6	84	20.9	6.306	1	**0.012**
Perceived economic status									
Lower	333	8.3	288	8.0	45	11.2	14.190	2	**<0.001**
Fair	2,776	69.6	2,481	69.2	295	73.4			
Higher	880	22.1	818	22.8	62	15.4			
Depression (CESD-10 ≥ 10)	1,120	28.1	938	26.1	182	45.3	65.466	1	**<0.001**

	Mean	SD	Mean	SD	Mean	SD	*Z*	df	*p*
Age (years)	81.80	10.42	80.64	9.91	92.20	8.98	20.006	-	**<0.001**
IADL Total	19.66	5.42	20.45	4.84	12.62	5.24	−23.833	-	**<0.001**
Sleep duration (hours per day)	7.23	2.19	7.18	2.11	7.65	2.78	2.916	-	**0.004**

Bolded values: <0.05; df, degree of freedom; SD, standard deviation; CESD-10, 10-item Center for Epidemiologic Studies Short Depression Scale; IADL, Instrumental Activities of Daily Living.

### 3.2 Prevalence of depression and cognitive impairment

The overall prevalence of depression (CESD-10 total score ≥ 10) among older adults with hypertension was 28.1% (*n* = 1,120, 95%CI = 26.7–29.5%), while the prevalence of cognitive impairment was 10.1% (*n* = 402; 95% CI = 9.2–11.1%).

### 3.3 Correlates of cognitive impairment

**Figure 1:**
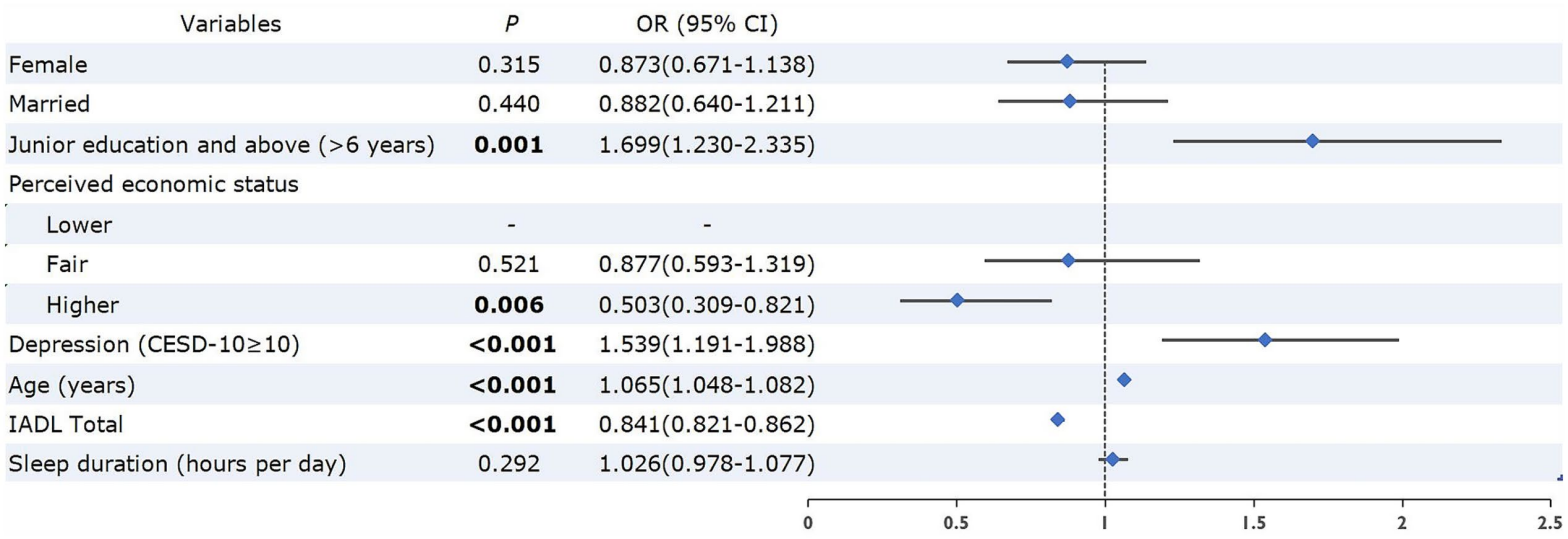
Independent correlates of cognitive impairment among older adults with hypertension. Bolded values: <0.05. OR, odds ratio; CI, confidence interval; CESD-10, 10-item Center for Epidemiologic Studies Short Depression Scale; IADL, Instrumental Activities of Daily Living.

Univariable analyses (Table 1) showed that participants with cognitive impairment were more likely to suffer from depression (45.3% vs. 26.1%, *p* < 0.001) and have longer sleep duration [7.65 (SD = 2.78) hours vs. 7.18 (SD = 2.11) hours per day, *p* = 0.004] compared to the non-cognitive impairment group. After controlling for confounders with logistic regression analysis, participants with depression (OR = 1.539, *p* < 0.001) had a significantly higher risk of cognitive impairment, while no significant link between sleep duration and cognitive impairment was found. Additionally, participants with higher education level (OR = 1.698, *p* = 0.001) and older age (OR = 1.064, *p* < 0.001) had a significantly higher risk of cognitive impairment, while those with better physical function had a lower risk of cognitive impairment (OR = 0.841, *p* < 0.001) (Figure 1).

### 3.4 Nonlinear association between depression, sleep duration and cognitive impairment

**Figure 2:**
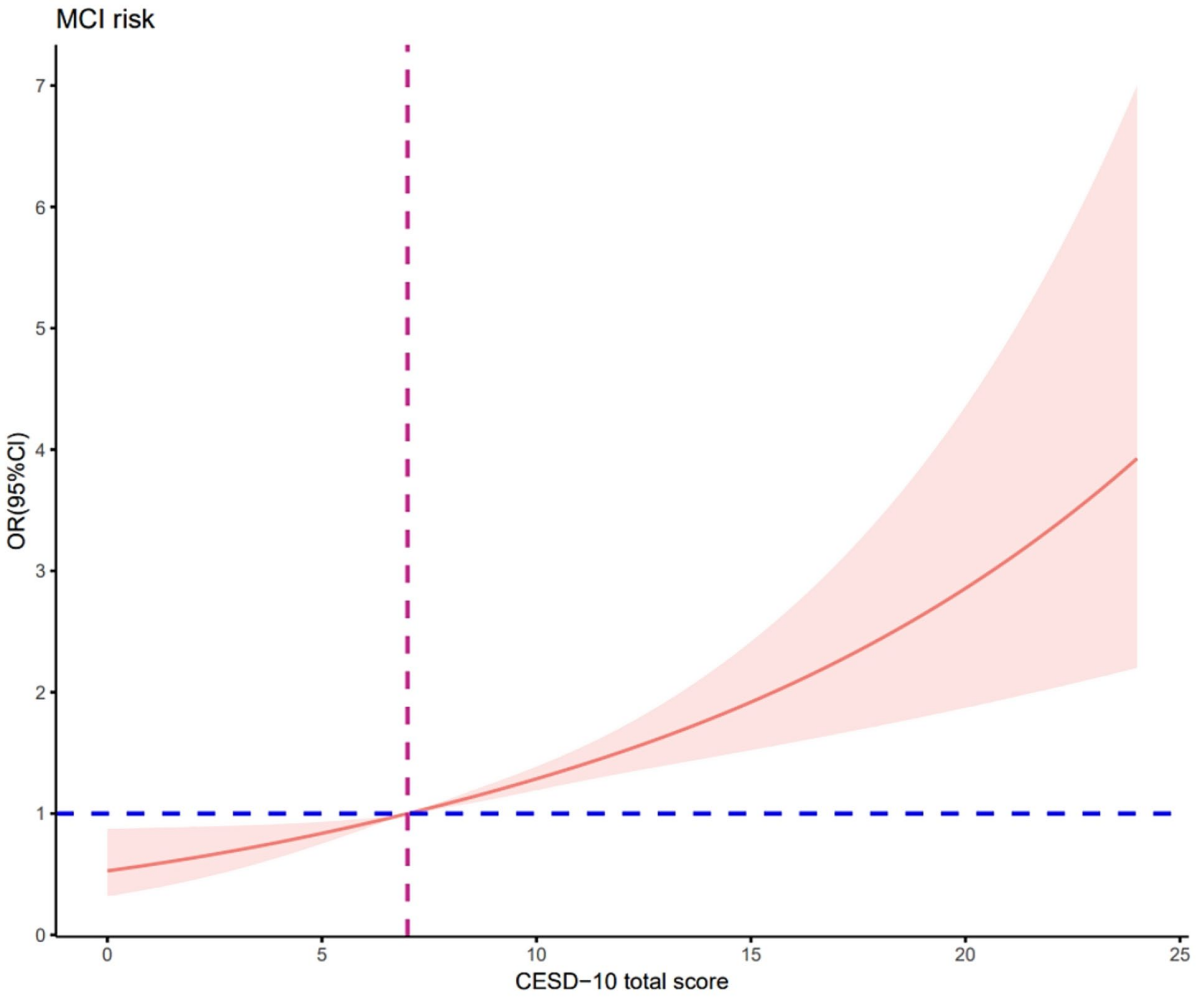
Restricted cubic spline regression of CESD-10 score and cognitive impairment risk. Data were fitted by a 3-knotted restricted cubic spline logistic regression model, adjusted for age, education level and IADL. Nonlinear test: *χ*^2^ =0.06, df = 1, *p* = 0.814; OR = 1, CESD-10 total score = 7.0.

**Figure 3:**
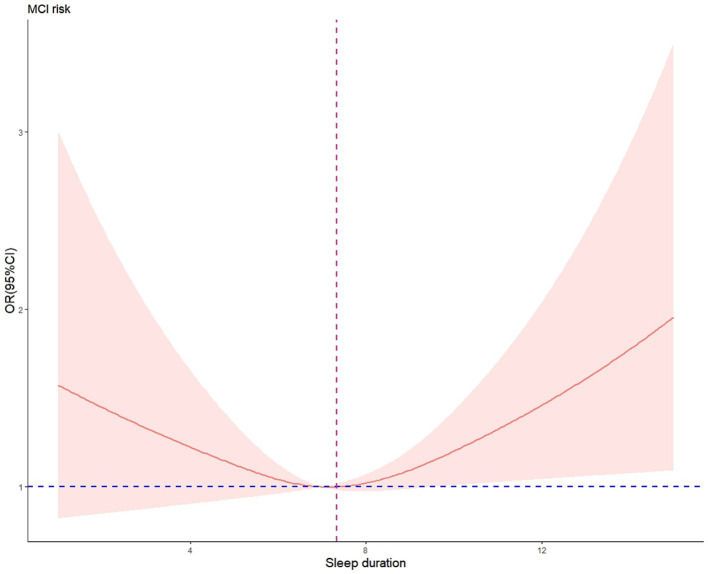
Nonlinear association of sleep duration and cognitive impairment risk. Data were fitted by a 3-knotted restricted cubic spline logistic regression model, adjusted for age, education level, IADL and depression. Nonlinear test: *χ*^2^=4.19, df = 1, *p* = 0.041; OR = 1, sleep duration = 7.3.

**Figure 4:**
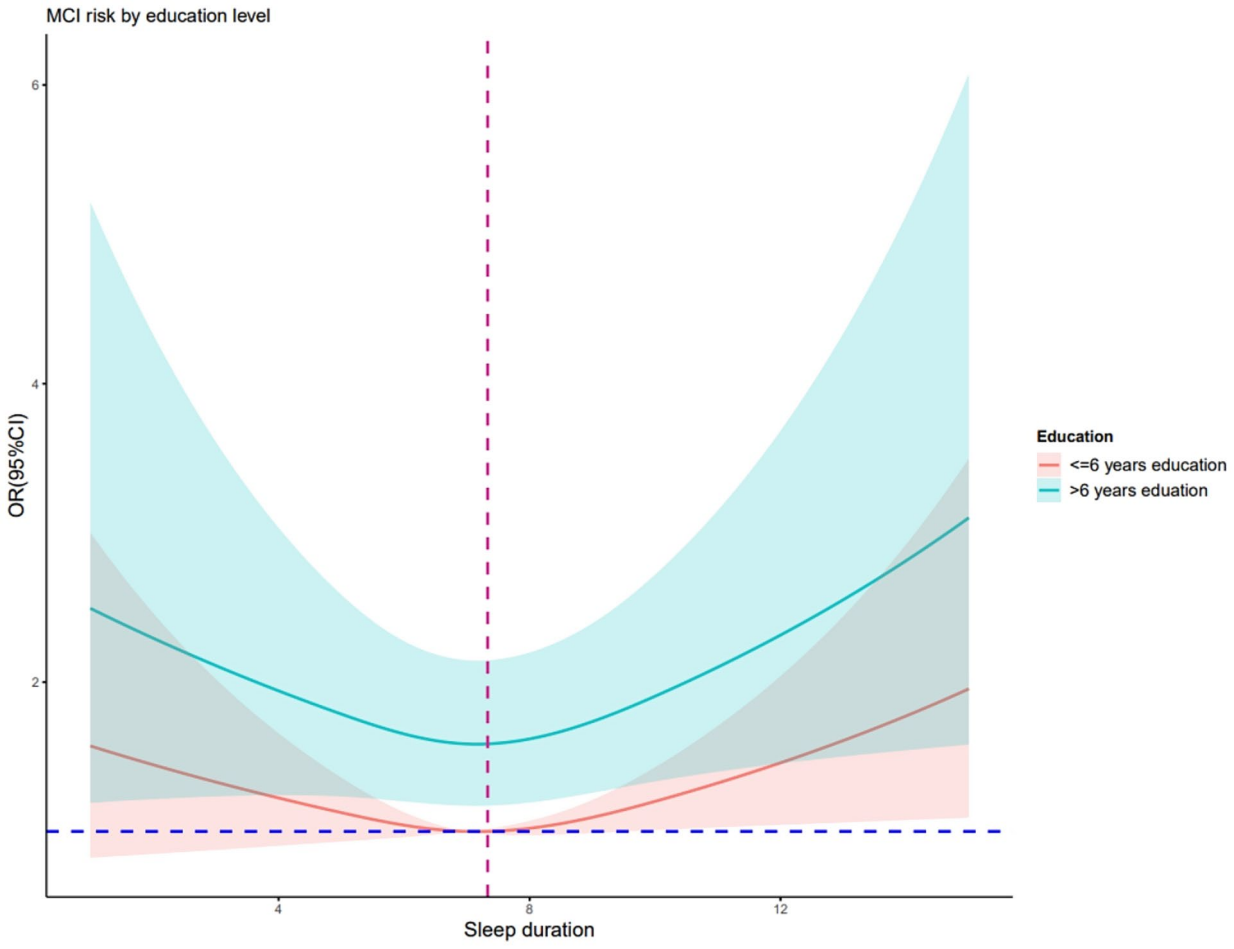
Nonlinear association of sleep duration and cognitive impairment risk regarding different education years Data were fitted by a 3-knotted restricted cubic spline logistic regression model, adjusted for age, education level, IADL and depression.

After adjusting for confounding factors, RCS regression demonstrated that there was a linear association between depression and cognitive impairment risk (nonlinear *p* = 0.814, Figure 2), but a U-shaped nonlinear association existed between sleep duration and cognitive impairment risk (*p* = 0.040, Figure 3). Both shorter (<6.6 h) and longer (>7.7 h) sleep duration per day were associated with higher cognitive impairment risk, with an inflection point at 7.3 h per day. Figure 4 illustrates the nonlinear relationship by different education levels. The OR values of participants with over 6 years of education were generally higher than those of participants with lower education level, indicating that for participants with a higher education level, the effect of sleep duration on cognitive impairment risk might be more significant.

## 4 Discussion

This was the first study to examine the nonlinear associations between depression, sleep and cognitive impairment in a hypertensive older adult population.

### 4.1 Prevalence of cognitive impairment and depression

In this study, the prevalence of cognitive impairment was 10.1% (95% CI = 9.2–11.1%), which is lower than the results of a meta-analysis in China that found pooled prevalence rates of 17% (95% CI = 15–19%) in community-based hypertensive patients and 19% (95% CI = 15–23%) in the subgroup using MMSE scale (Qin et al., 2021). In another study, the prevalence of cognitive impairment among hypertensive adults aged 55 and older in China was 19.1% (95% CI = 19–25.3%) (Deng et al., 2021). One possible reason for the lower prevalence observed in this study was that the cognitive impairment was defined using education-specific cutoffs. As our sample exhibited generally low education levels, therefore, most of the sample in this study defined cognitive impairment using cutoff scores of <18 and ≤20, rather than scores of ≤24 used in other studies. Moreover, a previous study (Cui et al., 2011) found that in a low-education Chinese population the prevalence of cognitive impairment screening with the education-specific cutoff scores was lower than that with the scores of 23/24 cutoff (7.0% vs. 35.6%), thus supporting the reliability of the education-specific cutoff scores.

The prevalence of depression in this study was 28.1% (95%CI = 26.7–29.5%), which is line with prevalence of 28% (95%CI = 20–36%) found in a meta-analysis of studies among older adults with hypertension across six countries (Gan et al., 2023). However, our finding was lower than that of the subgroup analysis for China (35%, 95%CI = 29–41%) but higher than the prevalence of the other included countries (16%, 95%CI = 8–26%). Differences across studies might be due to variations in study design such as sampling methods, sample sizes and assessment tools as well as the characteristics of the study samples.

### 4.2 Linear relationship between depression and cognitive impairment

This study found that hypertensive older adults with depressive symptoms had a higher risk of cognitive impairment (OR = 1.539, 95%CI = 1.191–1.988), which is consistent with findings from previous cross-sectional and cohort studies in older adults showing that severity of depression is positively associated with significant cognitive decline (Muhammad and Meher, 2021; Liu et al., 2023; Wei et al., 2019; Zhou et al., 2021). For instance, a cross-sectional study (Zhou et al., 2021) demonstrated that depression severity among older adults was negatively correlated with cognitive performance across all dimensions (i.e., orientation, memory, attention and calculation, as well as language) as measured by MMSE score. After controlling for age, gender and comorbidities, previous research also found that the combination of hypertension and depression substantially raises the risk of cognitive impairment (OR = 2.02, 95%CI = 1.60–2.54) (Scuteri et al., 2011b). Similarly, a retrospective cohort study revealed that older women with cumulative depressive symptoms had a two-fold increased risk of cognitive impairment and dementia (Zeki Al Hazzouri et al., 2014).

Furthermore, we identified a linear relationship between the CESD-10 total score and cognitive impairment risk, which aligns with another study that used the CLHLS data to explore such nonlinear relationship in older adults (Wu et al., 2024). Our results of RCS regression corroborated with those from logistic regression, reinforcing the finding that higher depression levels are associated with increased risk of cognitive decline in this population. In contrast, two studies using larger samples from the CLHLS (*N* = 13,840) and National Health and Nutrition Examination Survey (NHANES) respectively, identified a J-shaped nonlinear association between depression and cognitive impairment among older adults (Ding et al., 2024; Yao et al., 2024). This discrepancy might be due to the differences between older adults with hypertension and general older adult population. Additionally, variations in sample size, measurement tools and covariates included in the RCS regression could contribute to the different results. Therefore, it is essential to validate these findings in future research using larger samples of older adults with hypertension.

One possible mechanism linking depression and cognitive impairment is neurotransmitter disruption (e.g., dopamine and serotonin), which is closely associated with the development of certain mental health problems, such as depression, and can negatively impact cognitive function such as memory and executive functioning (Sierksma et al., 2010). Moreover, depressive symptoms frequently coexist with anxiety and psychological stress, which can further affect cognitive functioning negatively (Kraynak and Andreescu, 2022). These findings highlight the critical need for integrated mental health care for older adults with hypertension to address cognitive impairment. Interventions targeting depression not only can enhance mental health outcomes but also potentially alleviate cognitive decline.

### 4.3 U-shaped nonlinear association between sleep duration and cognitive impairment risk

Although binary logistic regression analysis revealed no linear association between sleep duration and cognitive impairment, RCS regression identified a significant U-shaped nonlinear association after adjusting for confounding factors, with both short and prolonged sleep duration linked to a higher risk of cognitive impairment. This finding is consistent with studies in older adults showing that both insufficient and excessive sleep could negatively impact cognitive function (Ma et al., 2020; Song et al., 2024), but is inconsistent with studies reporting a J-shaped relationship where only long sleep duration was associated with the risk of cognitive impairment among older adults (Zhang et al., 2022; Ding et al., 2024). In contrast, a meta-analysis found that both insufficient and excessive sleep duration increased the risk of cognitive decline among older adults (Lo et al., 2016), and another systematic review demonstrated a U-shaped dose–response relationship between sleep duration and cognitive impairment (Wu et al., 2018). Additionally, a previous longitudinal study found that both short and long sleep duration were linked to the onset of mild cognitive impairment (MCI) in middle-aged and older populations (Liu et al., 2022), which supports the findings of this study.

This study indicated that the ideal range for older adults with hypertension was 6.6 to 7.7 h of sleep per day, with 7.3 h per day being identified as having the lowest risk of cognitive impairment. Similarly, previous studies found that sleep duration less than 6 h or more than 8 h per day could negatively affect cognitive function (Song et al., 2024; Li et al., 2022). Specifically, older adults sleeping under 6 h per day faced a 30% higher dementia risk compared to those with typically 7-h sleep duration (Sabia et al., 2021).

We found that participants with higher education level (>6 years) had a high risk of cognitive impairment in logistic regression, which is inconsistent with previous findings that lower education was associated with MCI and dementia in older adults (Jones et al., 2024; Jia et al., 2020). Additionally, the U-shaped relationship between sleep duration and cognitive impairment risk was particularly evident in hypertensive older adults with higher education level, which suggests that individuals with higher education level might be more vulnerable to cognitive impairment risks associated with both insufficient and excessive sleep. These findings could be explained by cognitive reserve theory (Xu et al., 2015), which proposed that individuals with higher education levels might engage in more cognitively demanding activities, thus increasing their sensitivity to factors that disrupt cognitive resilience such as irregular sleep patterns. However, such interpretation should be treated with caution, and further research is needed to confirm such relationships.

### 4.4 Strengths and limitations

The main strengths of this study included the large, nationally representative sample and the use of RCS to explore nonlinear relationships between depression, sleep duration and cognitive impairment. However, several limitations should be noted. First, due to the cross-sectional study design, causal interpretations between depression, sleep duration and cognitive impairment could not be inferred. Second, both depression and sleep duration were based on self-report, which might result in recall bias, and the data in the CLHLS did not differentiate between nighttime sleep and daytime naps. Third, there are other confounding factors related to cognitive impairment (e.g., number and severity of physical comorbidities, hearing loss and air pollution) (Wang et al., 2025) which were not included in the analysis due to numerous missing or unavailable data in the CLHLS. Hence, various relevant risk factors should be included in future research. Fourth, although the data were collected in 2017–2018, the associations identified remain valuable for understanding symptom-level relationships in this population. Future studies are needed to validate these findings. Finally, the definition of hypertension in the Chinese guidelines was ≥140/90 mmHg during the 2017–2018 study period (Liu, 2020), which differed from the 2017 U.S. guideline (≥130/80 mmHg) (Whelton et al., 2018). This discrepancy might potentially limit direct comparisons with studies based on the international criteria.

## 5 Conclusion

The results of this study provide novel insights into the association between depression, sleep duration and cognitive impairment among hypertensive older adults. Both depression and suboptimal sleep duration were associated with increased risk of cognitive impairment. Individuals with higher educational levels were more vulnerable to the risk of cognitive impairment linked to irregular sleep patterns. Interventions for managing depressive symptoms and optimizing sleep duration, particularly for those with higher educational backgrounds could address the risk of cognitive impairment in older adults with hypertension.

## Data Availability

Publicly available datasets were analyzed in this study. This data can be found at: https://doi.org/10.18170/DVN/WBO7LK.
